# Association between bullying at school and tooth loss among 15–19-year-olds from southern Brazil

**DOI:** 10.1590/1807-3107bor-2024.vol38.0083

**Published:** 2024-09-13

**Authors:** Giovanna Leal KLEIN, Letícia Donato COMIM, Ângela DALLA NORA, Débora Nunes de Oliveira RACKI, Julio Eduardo do Amaral ZENKNER, Luana Severo ALVES

**Affiliations:** (a) Universidade Federal de Santa Maria – UFSM, Department of Restorative Dentistry, Santa Maria, RS, Brazil.; (b) Universidade Federal de Santa Maria – UFSM, Department of Stomatology, Santa Maria, RS, Brazil

**Keywords:** Tooth Loss, Bullying, Adolescent, Cross-Sectional Studies, Multilevel Analysis

## Abstract

This study aimed to investigate the association between bullying at school and tooth loss in southern Brazilian adolescents. This population-based cross-sectional study included a representative sample of 15–19-year-old students attending high schools in Santa Maria, southern Brazil. Data on sociodemographic and behavioral variables were collected through questionnaires. Contextual data on bullying at school was provided by educational institutions (bullying episodes in the previous year: ‘no,’ ‘sometimes,’ or ‘often’). Tooth loss was clinically assessed by the M component of the DMFT index, modeled as a discrete variable. Multilevel Poisson regression was used, and rate ratios (RR) and 95% confidence intervals (CI) were estimated. The prevalence of tooth loss was 9.2% (95%CI = 7.5–10.8). Adolescents who attended the schools where bullying events often occurred had 0.39 (95%CI = 0.33–0.45) missing teeth, on average, in contrast to an average of 0.14 (95%CI = 0.08–0.19) among those whose schools did not experience bullying in the previous year. After adjusting for important cofactors, the contextual variable of bullying at school remained significantly associated with the study outcome. Adolescents who attended schools where bullying frequently occurred were 2.49-fold more likely to have an additional missing tooth than those whose school did not experience bullying in the previous year (RR = 2.49, 95%CI = 1.37–4.51, p = 0.003). In conclusion, the frequent bullying episodes at school were associated with more permanent teeth lost due to caries in this population. Hence, improving the school environment may improve the oral health of adolescents.

## Introduction

Bullying has been described as an event in which a single person or a group of people repeatedly engage another individual in embarrassing situations of an offensive nature.^1^ Verbal bullying is the most common type of aggression in schools, especially among adolescents^2,3^, with a prevalence ranging from 14% to 72% in Brazil.^2,4^ It has been suggested that bullying can occur in different spheres of societal life. Economic disparities between schools and socioeconomic inequalities at the national and individual levels are associated with a higher prevalence of adolescent exposure to bullying.^5-7^

Considering the clinical aspect, tooth loss is the final consequence of severe dental caries, and the last national oral health survey indicated that it affected 17.4% of Brazilian adolescents aged 15 to 19 years.^8^ Early tooth loss in adolescence has been associated with extensive tooth loss in adulthood,^9^ thus warranting the investigation of this outcome in young populations.

It has been suggested that contextual factors at school, such as physical structure and social environment, may influence oral health. Previous studies have shown that healthier school environments (those more favorable to promoting health and safety) are associated with fewer cases of dental trauma,^10^ and lower prevalence^11^ and incidence^12^ of dental caries and tooth loss.^13^ Active health promotion actions addressing topics such as disease prevention, and using more accessible language for adolescents to understand the issue better, also seem to benefit the students’ oral health in the long term.^14,15^ In addition, schools with more favorable environments for promoting oral health have students who tend to visit the dentist more often, and consume soft drinks and sweets less often.^16,17^

Despite these previous findings, no study, to the best of our knowledge, has investigated the association between bullying at the school level and oral health outcomes, such as tooth loss. Therefore, the present study aimed to investigate the association between bullying at school and tooth loss among 15-19-year-old adolescents from southern Brazil. We hypothesized that adolescents attending schools where bullying occurs more often are more likely to have tooth loss.

## Methods

### Study Design and Sample

This population-based cross-sectional study was approved by the Research Ethics Committee of the Federal University of Santa Maria (number 2.178.299). All the institutions involved in the study agreed to perform it, and gave their required approval. All the patients or legal guardians were informed of the study purposes, and signed a written informed consent form. Students received a report of their oral health status, and were referred to dental treatment when needed.

Data collection was conducted in the city of Santa Maria, state of Rio Grande do Sul, southern Brazil. All 37 high schools in the municipality (26 public and 11 private) were invited to participate in the study. Adolescents born in 1999–2003, who were enrolled in the regular school year, and who attended any school period (morning, afternoon, or night) were considered eligible for the study. The sample did not include individuals using fixed orthodontic appliances or those presenting special needs.

A simple random sampling procedure was adopted in each school. Subjects were randomly selected in proportion to school size, using a table of random numbers (www.random.org). The sample size calculation used the following parameters: prevalence rate of 50% (worst case scenario), confidence interval (CI) of 95%, power of 80%, and precision level of 3%. It was estimated that 1,066 students would be needed for the study, and a non-participation rate of 50% was added, totaling 1,600 adolescents who would be invited to participate. This sample size far exceeded that needed for an association study, considering a prevalence of tooth loss of 17%^8^ and an association estimate of 2.

### Data Collection

Data collection was conducted from March to November 2018, and included questionnaires and a clinical examination. The socioeconomic and demographic questionnaire was previously tested and adjusted to allow better comprehension. Then, it was either sent to the parents/legal guardians of selected students aged <18 years, or applied to the students aged ≥ 18 years. This questionnaire contained questions on demographic information (sex, age, and skin color) and socioeconomic characteristics (mother’s education and socioeconomic status [SES]). SES was measured through questions assessing issues such as household goods and basic housing conditions.^18^

A second questionnaire was applied to students to collect data regarding behavioral variables (sugar-sweetened drink consumption, tooth brushing frequency, and dental care service). The educational institution provided contextual data on bullying at school through an on-site questionnaire filled out by the main person in charge of the institution. The question used was: “Was there any bullying event at this school in 2018?” and the possible answers were no, sometimes, or often. All schools answered this question at the beginning of 2019.

Clinical examination was performed by two previously calibrated examiners (ADN and DNOR), in a room provided by the school. First, gingivitis was assessed using the gingival bleeding index (GBI) in six sites per tooth.^19^ Then, the teeth were cleaned and dried to determine and record dental caries by using the decayed, missing, and filled teeth (DMFT) index under artificial light, with a clinical mirror and a WHO probe.^20^ Examiners were trained and calibrated for the DMFT index before beginning the study. The minimal value of the intraexaminer kappa coefficient was 0.81, and that of the interexaminer kappa coefficient was 0.80. As for the GBI, training was performed under the supervision of an experienced periodontist, but no calibration was performed due to the temporary nature of the condition.

### Data Analysis

The outcome of this study was tooth loss, defined as the number of permanent teeth lost due to caries or residual roots (M component of the DMFT index), which was modeled as a discrete variable. The main explanatory variable was bullying at school (no vs. sometimes or often, as previously described). Demographic variables were sex (male or female), age (< 17 years or ≥ 17 years), and skin color (white or non-white). Mother’s education was categorized as ≤primary school, high school, or university. Socioeconomic status (SES) used cut-off points proposed by the standard Brazilian economic classification.^18^ Households were classified as low SES (≤ 16 points, corresponding to social classes D and E), mid-low SES (≥ 17 to ≤ 22 points, corresponding to social class C2), mid-high SES (≥ 23 to ≤ 28 points, corresponding to social class C1), or high SES (≥ 29 points, corresponding to social classes A, B1, and B2). Behavioral variables included the consumption of sugar-sweetened drinks (≤ twice a day or ≥ 3 times a day), frequency of tooth brushing (≥3 times a day or ≤twice a day), and type of dental service accessed by the adolescents (private, public, or other). Lastly, clinical variables included gingivitis, dichotomized into absent (< 10% of sites with bleeding on probing) or present (≥ 10% of sites with bleeding on probing),^21^ and untreated caries, defined as the number of teeth with dentin cavities (D component of the DMFT index) and categorized into 0, 1–2, or ≥ 3.

Data analysis was performed using STATA software (Stata 14.2, Stata Corporation, College Station, USA) and survey commands that considered the survey design, including clustering, stratification, weighting, and robust variance estimation. A weight variable based on the probability of selection and population distribution according to sex and school type was used to adjust for the potential bias in the population estimates.^22^ A preliminary analysis comparing the mean number of missing teeth among the categories of explanatory variables was conducted using the Wald test.

Multilevel Poisson regression analysis assessed the association between the main explanatory variable (bullying at school) and the number of missing teeth. The multilevel model considered adolescents as the first-level unit and schools as the second level. The multilevel model used the scheme of fixed effects with a random intercept. In addition, the analysis was performed based on a contextual framework (Figure) adapted from the World Health Organization.^23^ Unadjusted and adjusted rate ratios (RRs) and 95% CIs were estimated. Adjusted estimates were controlled for sex, age, skin color, mother’s education, SES, sugar-sweetened drink consumption, tooth brushing frequency, dental care, gingivitis, and untreated caries, irrespective of their p-values.

**Figure:**
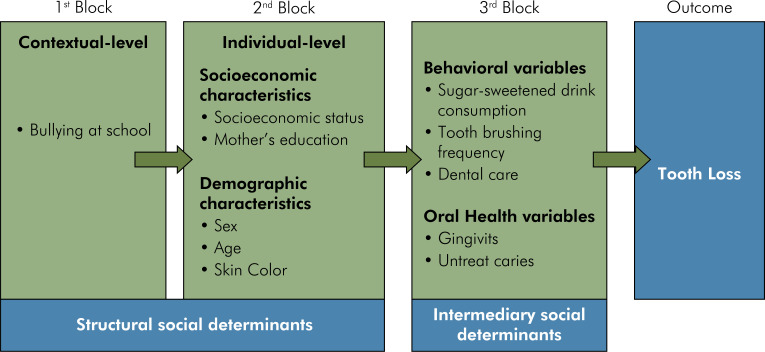
Theoretical model used to study the association between bullying at school and tooth loss, adapted from the World Health Organization.23

## Results

A total of 1,197 out of 1,656 individuals were included in the study, yielding a participation rate of 72.3%. The main reason for non-participation was refusal, given the lack of signed consent. Six schools refused to take part in the study. For this reason, the number of students to be selected in each school was adjusted proportionally in the 31 participating schools, to ultimately attain the needed sample size.

Table 1 shows the sample distribution and the mean number of missing teeth by explanatory variables. A total of 110 adolescents had at least one missing tooth, resulting in an overall tooth loss prevalence of 9.2% (95%CI: 7.5–10.8). The overall mean number of missing teeth in this population was 0.15 (0.11–0.18). The preliminary analysis showed a significantly higher number of missing teeth among adolescents who attended schools where bullying events often occurred (0.39; 95%CI: 0.33–0.45) than where there was no bullying (0.14; 95%CI: 0.08–0.19), or where bullying occurred sometimes (0.13; 95%CI: 0.09–0.17).

**Table 1:** Sample distribution and tooth loss by explanatory variables.

Variable	n (%)	Mean (95% CI)
Contextual variable		
Bullying at school*		
No	612 (51.8)	0.14 (0.08–0.19)^a^
Sometimes	512 (43.3)	0.13 (0.09–0.17)^a^
Often	58 (4.9)	0.39 (0.33–0.45)^b^
Individual variable		
Demographic and socioeconomic variable		
Sex		
Boys	513 (42.9)	0.14 (0.10–0.17)^a^
Girls	684 (57.1)	0.15 (0.10–0.21)^a^
Age (years)		
< 17	655 (54.7)	0.08 (0.43–0.11)^a^
≥ 17	542 (45.3)	0.23 (0.16–0.30)^b^
Skin color*		
White	779 (67.0)	0.11 (0.07–0.15)^a^
Non-white	384 (33.0)	0.22 (0.17–0.27)^b^
Mother’s education*		
University	192 (16.7)	0.03 (0.01–0.06)^a^
High school	380 (33.1)	0.13 (0.08–0.18)^b^
≤ Primary school	577 (50.2)	0.19 (0.13–0.25)^b^
Socioeconomic status*		
High	335 (28.9)	0.05 (0.02–0.09)^a^
Mid-high	302 (26.1)	0.14 (0.06–0.23)^ab^
Mid-low	320 (27.6)	0.20 (0.14–0.26)^b^
Low	201 (17.4)	0.25 (0.12–0.39)^b^
Behavioral variable		
Sugar-sweetened drink consumption*		
≤ twice a day	664 (55.6)	0.11 (0.07–0.14)^a^
≥ 3 times a day	531 (44.4)	0.19 (0.14–0.25)^b^
Tooth brushing frequency*		
≥ 3 times a day	566 (47.4)	0.11 (0.08–0.15)^a^
≤ Twice a day	628 (52.6)	0.17 (0.12–0.23)^b^
Dental care service*		
Private	642 (57.8)	0.13 (0.08–0.18)^a^
Public	289 (26.0)	0.24 (0.16–0.32)^b^
Other	180 (16.2)	0.09 (0.02–0.15)^a^
Oral health variable		
Gingivitis		
Absent (< 10% bleeding sites)	1,031 (86.1)	0.14 (0.10–0.18)^a^
Present (≥10% bleeding sites)	166 (13.9)	0.16 (0.09–0.24)^a^
Untreated caries (teeth)		
0	908 (75.9)	0.08 (0.05–0.11)^a^
1–2	247 (20.6)	0.28 (0.19–0.37)^b^
≥ 3	42 (3.5)	0.79 (0.50–1.07)^c^
Total	1,197 (100)	0.15 (0.11–0.18)

*Missing data; CI: Confidence interval; different letters indicate statistically significant differences among categories (p < 0.05, adjusted Wald test).

Table 2 presents the association between contextual- and individual-level explanatory variables and tooth loss. After adjusting for important cofactors, the contextual variable of bullying at school remained significantly associated with the study outcome. Adolescents who attended schools where bullying frequently occurred were 2.49-fold more likely to have an additional missing tooth than those whose schools did not record bullying in the previous year (RR = 2.49, 95%CI: 1.37–4.51, p = 0.003). In addition, the variables of age ≥ 17 years, non-white skin color, low SES, low maternal education, increased consumption of sugar-sweetened drinks, reduced tooth brushing frequency, and untreated caries were also associated with tooth loss in this population.

**Table 2:** Association between exploratory variables and number of missing teeth in adolescents (unadjusted and adjusted multilevel Poisson regression analysis).

Variable	Unadjusted		Adjusted	
	RR (95%CI)	p-value	RR (95%CI)	p-value
Contextual variable				
Bullying at school				
No	1.00		1.00	
Sometimes	0.99 (0.47–.2.07)	0.98	0.81 (0.54–1.21)	0.31
Often	3.49 (0.98–12.35)	0.05	2.49 (1.37–4.51)	0.003
Individual variable				
Demographic and socioeconomic variable				
Sex				
Boys	1.00		1.00	
Girls	1.14 (0.85–1.53)	0.39	1.03 (0.74–1.43)	0.85
Age (years)				
< 17	1.00		1.00	
≥ 17	2.55 (1.85–3.51)	< 0.001	2.15 (1.51–3.05)	<0.001
Skin color				
White	1.00		1.00	
Non–white	1.65 (1.22–2.22)	0.001	1.58 (1.14–2.19)	0.006
Mother’s education				
University	1.00		1.00	
High school	3.26 (1.46–7.30)	0.004	4.72 (1.44–15.4)	0.01
≤ Primary school	3.99 (1.80–8.84)	0.001	3.59 (1.08–11.9)	0.04
Socioeconomic status				
High	1.00		1.00	
Mid–high	2.59 (1.50–4.48)	0.001	2.28 (1.25–4.13)	0.007
Mid–low	2.99 (1.75–5.11)	< 0.001	2.66 (1.46–4.84)	0.001
Low	3.52 (2.00–6.18)	< 0.001	2.96 (1.55–5.65)	0.001
Behavioral variable				
Sugar–sweetened drink consumption				
≤ twice a day	1.00		1.00	
≥ 3 times a day	1.67 (1.24–2.25)	0.001	1.55 (1.12–2.15)	0.009
Tooth brushing frequency				
≥ 3 times a day	1.00		1.00	
≤ Twice a day	1.35 (1.00–1.83)	0.05	1.63 (1.16–2.28)	0.005
Dental care				
Private	1.00		1.00	
Public	1.46 (1.06–2.02)	0.02	1.13 (0.79–1.61)	0.49
Other	0.58 (0.34–0.98)	0.04	0.64 (0.37–1.09)	0.10
Oral health variable				
Gingivitis				
Absent (< 10% bleeding sites)	1.00		1.00	
Present (≥10% bleeding sites)	1.12 (0.75–1.68)	0.58	0.94 (0.60–1.48)	0.80
Untreated Caries				
0	1.00		1.00	
1–2 teeth	3.35 (2.42–4.62)	< 0.001	2.51 (1.76–3.56)	< 0.001
≥ 3 teeth	7.64 (4.99–11.7)	< 0.001	4.71 (2.87–7.73)	< 0.001

CI: Confidence interval; RR: rate ratio.

## Discussion

This study raised the hypothesis that adolescents who attended schools with more bullying episodes were more likely to have more missing teeth. Our results confirmed this hypothesis, since we found that the mean number of missing teeth was significantly higher among adolescents who attended schools where bullying occurred frequently. This association remained significant even after adjusting for several factors that could influence this relationship. To the best of our knowledge, this is the first study to address the possible association between bullying at the school level and adolescent tooth loss.

Bearing in mind the contextual nature of our main explanatory variable of bullying at school, we should discuss the influence of a safe and healthy school environment on students’ lives. Previous studies have reported that the school context is associated with oral health outcomes.^10-13,16,17^. Schools more favorable to promoting health and safety have students who tend to visit the dentist more often,^16^ and have fewer cases of dental trauma^10^ and dental caries.^11-12^ In Ontario, schools participating in the “Healthy Schools” program had a significantly lower percentage of children with decayed teeth and urgent dental treatment needs than non-participating schools.^24^ Furthermore, the study by Edasseri et al.^12^ points out that school environments that promote healthy eating habits, and that incorporate socioenvironmental and political aspects of health promotion can be particularly effective in reducing dental caries. In the present study, we observed that individuals who attended schools where bullying occurred often had more missing teeth than those whose schools had not experienced bullying in the previous year, thus suggesting that an unhealthy school context may affect adolescents’ oral health. To the best of the authors’ knowledge, only one previous study investigated the association between school-level variables and tooth loss among adolescents.^13^ The authors showed that individuals attending schools that promote greater incentives for educational aspirations and professional growth have fewer missing teeth, thus suggesting that adolescents are more motivated to invest in the future, and have a greater awareness of oral health.^13^

In our study, the overall prevalence of tooth loss among 15–19-year-old adolescents was 9.2%, approximately half of the national mean (17.4%), according to the last national oral health survey.^8^ Considering global parameters, the general prevalence (for all ages) of severe tooth loss (≤ 9 remaining permanent teeth) in Brazil is significantly higher (3.9%) than the world average (2.4%).^25^ Even though our prevalence could be considered low considering national standards, the prevalence of tooth loss in the studied population deserves attention, especially considering the reduced age-related time of exposure to dental caries. Tooth loss^26^ and oral health^27^ may impact the quality of life of adolescents, thus putting into evidence the importance of this health context for the general well-being of young people. In addition, it has been suggested that early tooth loss may play a role in edentulism in the adolescent’s future.^9^

The association between both socioeconomic and behavioral factors and tooth loss has been studied previously, but only a few studies have investigated this age group. In the present study, adolescents aged 17 years or older, of non-white skin color, with lower SES and lower maternal education, had more missing teeth than their counterparts. Variables such as income, skin color, and age have been previously associated with higher rates of tooth loss.^8,28-30^ It has been shown that individuals with lower SES tend to have worse oral hygiene, consume more sucrose, and seek preventive dental care less frequently than individuals with higher SES.^31-33^ In our study, the high consumption of sugary drinks, lower tooth brushing frequency, and clinical factor of untreated caries were also associated with a higher number of missing teeth, in agreement with previous studies.^34-36^ Altogether, these results point to a greater extent and severity of dental caries (and consequently tooth loss) in less favored groups, reinforcing inequality in the distribution of the disease.^37^ This accounts for the negative influence of a poor socioeconomic context on the oral health outcomes observed in our study, as previously demonstrated in parameters of dental caries^38^ and gingival bleeding.^39^

Some limitations of the present study should be considered, such as its cross-sectional design, which does not allow measuring long-term impacts or addressing their causal relationships; however, this study design is appropriate to estimate the prevalence of outcomes, and create a hypothesis to be tested in future cohort studies. It could be argued that the contextual nature of the main explanatory variable, obtained using data from school reports, could be seen as a possible shortcoming of our study, and that the analysis of the individual perception of adolescents would provide more meaningful information. Nevertheless, our focus on studying the school environment enabled adopting a multilevel analysis to improve our understanding of the possible relationship between the school-level contextual factor of bullying and tooth loss in these individuals. Regarding the strengths of this study, we could emphasize its pioneering aspect, since it addresses a research question that has not been investigated previously, and that uses data from a large representative sample of the population, thus improving the external validity of our findings.

In conclusion, this population-based cross-sectional study showed that the frequent occurrence of bullying episodes at school was associated with a higher number of permanent teeth lost due to caries in this population, a fact that suggests the detrimental effect of a stressful school environment on oral health.
